# Enhanced bioavailability and short-term safety of a micellar green tea extract formulation co-delivered with N-acetylcysteine: a randomized crossover study in healthy adults

**DOI:** 10.3389/fnut.2026.1940156

**Published:** 2026-09-23

**Authors:** Yiming Zhang, Afoke Ibi, Yun Chai Kuo, Peony Do, Min Du, Yoon Seok Roh, Roland Gahler, Mary Hardy, Simon Wood, Chuck Chang, Julia Solnier

**Affiliations:** 1 ISURA, Clinical Research, Burnaby, BC, Canada; 2 Factors Group R&D, Burnaby, BC, Canada; 3 Academy of Integrative and Holistic Medicine, San Diego, CA, United States; 4 Food, Nutrition and Health Program, University of British Columbia, Vancouver, BC, Canada; 5 School of Public Health, Faculty of Health Sciences, Curtin University, Perth, WA, Australia; 6 InovoBiologic Inc., Calgary, AB, Canada

**Keywords:** bioavailability, catechins, epigallocatechin gallate, green tea extract, GTNAC, lipid-based delivery, LipoMicel, N-acetylcysteine

## Abstract

**Introduction:**

Green tea catechins, particularly epigallocatechin gallate (EGCG), the principal polyphenol in green tea (*Camellia sinensis*), possess diverse bioactivity but suffer from poor absorption and rapid metabolism, leading to very limited oral bioavailability. To address these challenges, this study investigated the pharmacokinetics, safety, and tolerability of LipoMicel^®^ GTNAC^®^ (LGT), a novel lipid-based green tea extract formulation co- delivered with N-acetylcysteine (NAC), compared with a standard green tea formulation (SGT) in healthy adults.

**Methods:**

The study comprised two parts: a randomized, double-blind, crossover pharmacokinetic trial in 16 healthy adults comparing LGT 330 mg with SGT 330 mg and SGT 660 mg, followed by a 30-day open-label, single-arm safety study evaluating LGT 1,320 mg/day in 13 participants.

**Results:**

For EGCG, LGT significantly increased baseline-adjusted systemic exposure compared with dose-matched SGT, producing a 4.2-fold higher iAUC₀–₂₄ (1857 ± 1,479 vs. 443 ± 1,570 ng·h/mL; *p* = 0.032) and a 3.0-fold higher iC_max_ (267 ± 235 vs. 88.6 ± 60.0 ng/mL; *p* = 0.037). No clinically significant adverse events or clinically meaningful changes in blood chemistry were observed during the 30-day safety phase.

**Conclusion:**

These findings demonstrate that LGT significantly enhances systemic EGCG exposure while remaining well tolerated for over 30 days in healthy adults. Larger and longer-term studies are warranted to determine whether improved EGCG exposure translates into clinically meaningful health benefits.

**Clinical trial registration:**

https://clinicaltrials.gov/, registration number NCT06971536.

## 1 Introduction

Green tea, made from *Camellia sinensis*, has been consumed for centuries in many Asian countries and is renowned for its health benefits (1, 2). It is rich in bioactive phytochemicals, particularly catechins, as well as other polyphenols, flavonoids, and theanine, which have been associated with antioxidant, anti-inflammatory and metabolic health effects (3, 4). Regular green tea consumption has been linked to reduced risks for various cancers, cardiovascular diseases, and high blood pressure, while also exhibiting antimicrobial and weight management benefits (5, 6).

Green tea extract (GTE) is often used in dietary supplements and standardized to contain high levels of catechins (5, 7). The primary catechins include epicatechin (EC), epicatechin gallate (ECG), epigallocatechin (EGC), and the most abundant, bioactive and potent form, epigallocatechin gallate (EGCG) (1, 8). EGCG has well-documented anti-proliferative, anti-inflammatory, and antioxidant effects mediated through modulation of multiple oncogenic signaling pathways, epigenetic regulation via DNA methyltransferase (DNMT) and histone deacetylase (HDAC) inhibition, and direct induction of apoptosis across numerous cancer cell types (9–16).

Despite their benefits, catechins typically have low oral bioavailability due to poor absorption, extensive first-pass metabolism, and limited permeability (17). Human studies reported oral bioavailability as low as 1.68%, compared to other animals such as mice which showed a higher absorption range of 13–26.5% (18–20). The restricted uptake is partly attributed to an extensive hydration shell resulting from the catechins’ abundant hydroxyl groups (Figures 1, 2), which impart high hydrophilicity and low lipophilicity (21, 22). Consequently, catechin absorption largely occurs via passive diffusion rather than active transporter mechanisms (23–25).

**Figure 1:**
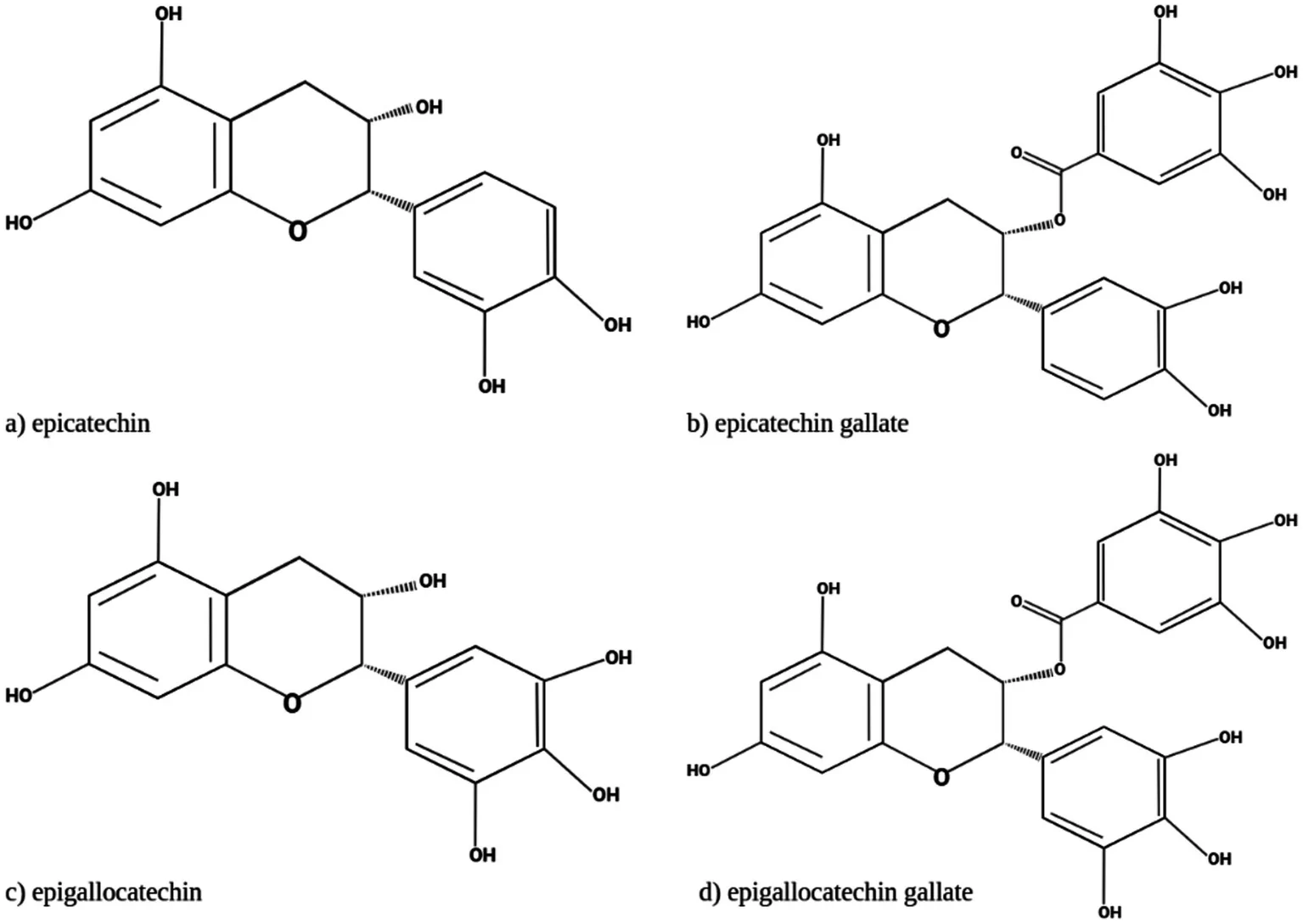
Parent green tea catechins epicatechin **(a)**, epicatechin gallate **(b)**, epigallocatechin **(c)**, and epigallocatechin gallate **(d)**. Created in BioRender. Ibi, A. (2026), https://BioRender.com/dtl936r.

**Figure 2:**
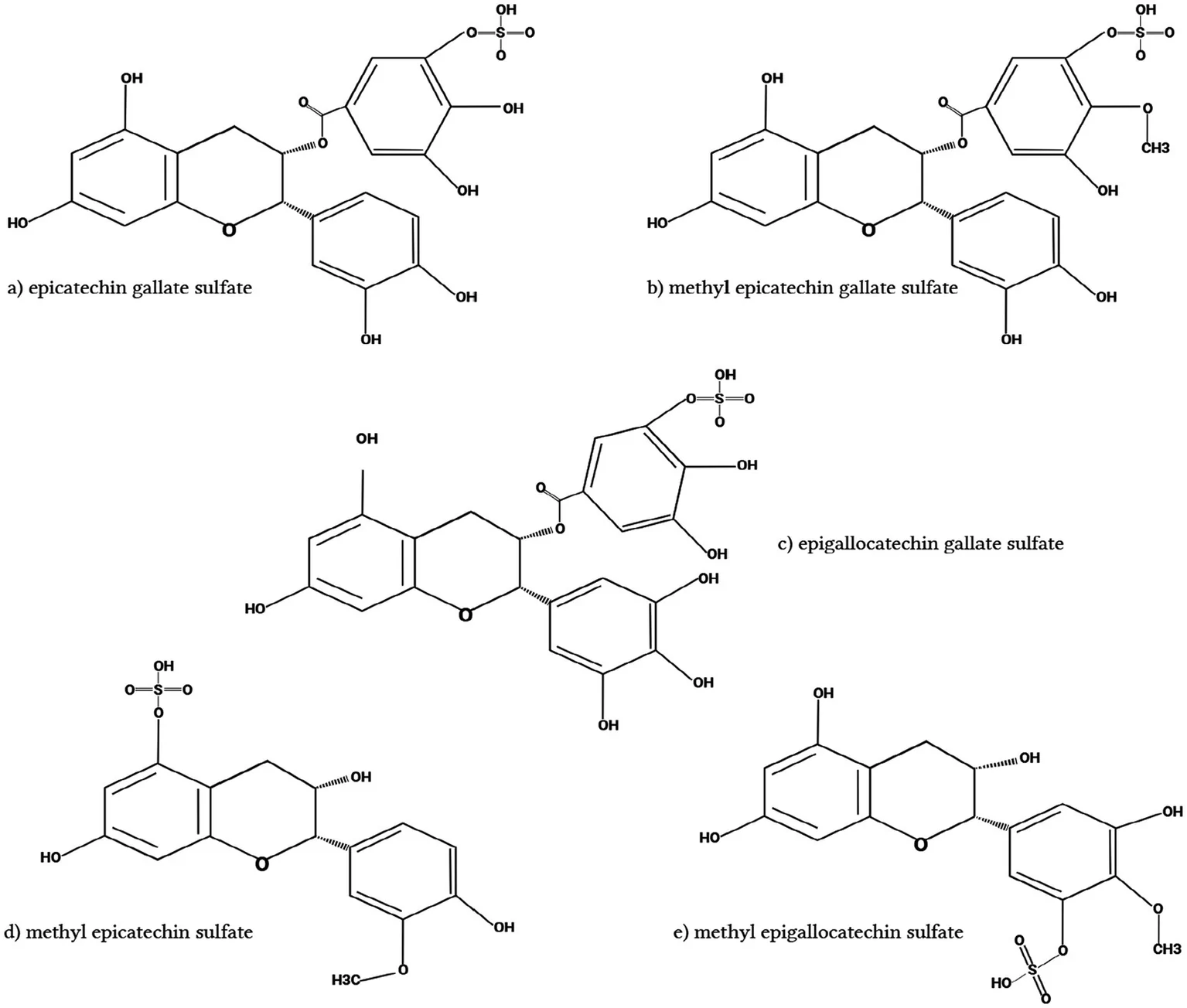
Green tea catechin metabolites epicatechin gallate sulfate **(a)**, methyl epicatechin gallate sulfate **(b)**, epigallocatechin gallate sulfate **(c)**, methyl epicatechin sulfate **(d)**, methyl epigallocatechin sulfate **(e)**. Created in BioRender. Ibi, A. (2026), https://BioRender.com/vsncrv6.

When orally consumed, catechins undergo extensive conjugation reactions such as sulfation, glucuronidation, and methylation (26). Sulfation is the dominant pathway in humans and produces circulating metabolites that can reach blood concentrations comparable to their parent catechins and may remain longer in circulation due to their increased stability (27, 28). Methylation also produces circulating metabolites that tend to persist longer in the bloodstream than their parent compounds, although this does not necessarily result in greater overall exposure (29–31). The presence of both these metabolites, therefore, can be key markers of absorption and systemic exposure. Measuring conjugated metabolites along with the parent compound may provide a more comprehensive and physiologically relevant picture of systemic exposure (32, 33).

While green tea as a beverage is generally recognized as safe, catechin-rich extracts have been associated with rare instances of gastrointestinal discomfort and hepatotoxicity when ingested as large bolus doses (34, 35). Emerging evidence suggests that these effects may be dose-dependent, with substantial inter-individual variability in susceptibility, and may also occur due to genetic differences (36), co-consumption with medications (37), or fasting conditions (38).

To better control the redox-dependent bioactivity of green tea catechins, a proprietary combination (GTNAC^®^) of green tea co-formulated with N-acetylcysteine (NAC), a well characterized antioxidant and glutathione precursor, was developed. NAC has been shown to support cellular antioxidant defenses and mitigate hepatic oxidative stress in various models (39–41). Its inclusion in this novel formulation was therefore intended to strengthen the overall antioxidant balance while also further strengthening the safety profile of high-bioavailability GTE catechins, particularly under conditions of sustained use or elevated exposure.

This study presents a comprehensive evaluation of the pharmacokinetics and safety of LipoMicel^®^ GTNAC (LGT), a novel formulation designed to enhance bioavailability and improve systemic delivery of catechins. Given that formulation strategies can significantly influence both absorption and tolerability, this study aimed to characterize the pharmacokinetic profile of LGT and evaluate its safety under high-dose conditions in human participants. The findings will inform the potential of LGT as a new Natural Health Product (NHP) or dietary supplement and provide a foundation for future research focused on optimizing green tea’s clinical utility in health promotion.

## 2 Materials and methods

### 2.1 Ethics and regulatory approvals

The study protocol and amendments were reviewed by the Canadian Shield Ethics Review board with OHRP Registration IORG0003491, FDA Registration IRB00004157, and approved on June 17, 2024, under REB Tracking Number: 2022-11-002. The study was conducted in accordance with the ethical principles in the Declaration of Helsinki. Written informed consent was obtained from participants upon enrollment. This study was registered on May 6, 2025, at ClinicalTrials.gov with clinical trial registration number NCT06971536.

### 2.2 Treatments

The following oral formulations of green tea extracts (*Camellia sinensis*) were investigated regarding their pharmacokinetics at a daily dose of 1–2 capsule per day; all capsules were stored at room temperature, protected from heat, moisture, and direct light.

LipoMicel^®^ GTNAC^®^ (Natural Factors, Burnaby, British Columbia, Canada) containing: 330 mg green tea extract and 240 mg N-Acetylcysteine as medicinal ingredients. Excipients include medium chain triglycerides, lecithin, xylitol, methylsulfonylmethane (MSM), peppermint flavor, and stevia. From herein referred to as “LGT.”

Unformulated Standard Green Tea Extract (Vancouver, British Columbia, Canada) containing: 330 mg green tea extract and 240 mg N-Acetylcysteine as medicinal ingredients and no excipients. From herein referred to as “SGT.”

### 2.3 Objectives

The primary objective of this study was to compare the pharmacokinetic profile of green tea catechins delivered via the LipoMicel^®^ formulation to a standard green tea extract formulation (SGT).

The secondary objective was to assess the safety and tolerability of the newly developed LGT formulation following daily supplementation at a high dose (1,320 mg/day) over 30 days. Safety was evaluated through clinical laboratory parameters.

### 2.4 Study participants

#### 2.4.1 Inclusion criteria

Participants were required to be healthy male or females aged 21–65 years. They were also required to fill out an online health questionnaire on their medical history upon study enrollment and provide voluntary informed written consent to participate in the study.

#### 2.4.2 Exclusion criteria

Participants with the following conditions were excluded:

- Use of medications such as anti-inflammatory or non-steroidal anti-inflammatory drugs.
- Medical history with any acute or chronic diseases such as cardiovascular, liver, or kidney disease.
- Use of substances such as alcohol, cannabis, nicotine, or tobacco
- Participation in another investigational study.
- Pregnancy
- Consumption of tea - particularly green tea and matcha.
- An unlimited daily intake of coffee (> 2 cups per day) and caffeine products (e.g., energy drinks, colas, large amounts of chocolate).

### 2.5 Study design

The study took place in November 2025 at the ISURA Research facility in Burnaby, British Columbia, Canada. This study consisted of two parts: a randomized, double-blind, crossover bioavailability study, followed by a 30-day open-label, single-arm safety evaluation. To reduce potential bias, treatment allocation for the crossover pharmacokinetic phase was performed by an independent study assistant, while participants, study coordinator, and principal investigator remained blinded to treatment identity during data collection. Outcomes were based on objective Liquid Chromatography-High Resolution Mass Spectrometry (LC-HRMS) and blood chemistry measurements, and statistical analyses were conducted using predefined methods. Data interpretation was reviewed by authors with scientific and clinical expertise, including contributors who were not involved in product formulation. Potential conflicts of interest are disclosed separately.

The first part of the study was a block randomized double-blind crossover trial designed to compare the pharmacokinetics of LipoMicel^®^ GTNAC^®^ (LGT) and a standard formulation (SGT). Each intervention phase lasted 24 h and was separated by a 1-week washout period (see Figure 3). Participants received a single capsule of the assigned formulation with approximately 200 mL of water, alongside a standardized breakfast. A third intervention was done with a double dose of the SGT. Capillary whole blood samples were collected at the following time points: baseline (0 h), 0.5, 1, 2, 3, 4, 6, 8, 10, 12, and 24 h post-dose. The final sample (24 h) was collected after an overnight fast.

**Figure 3:**
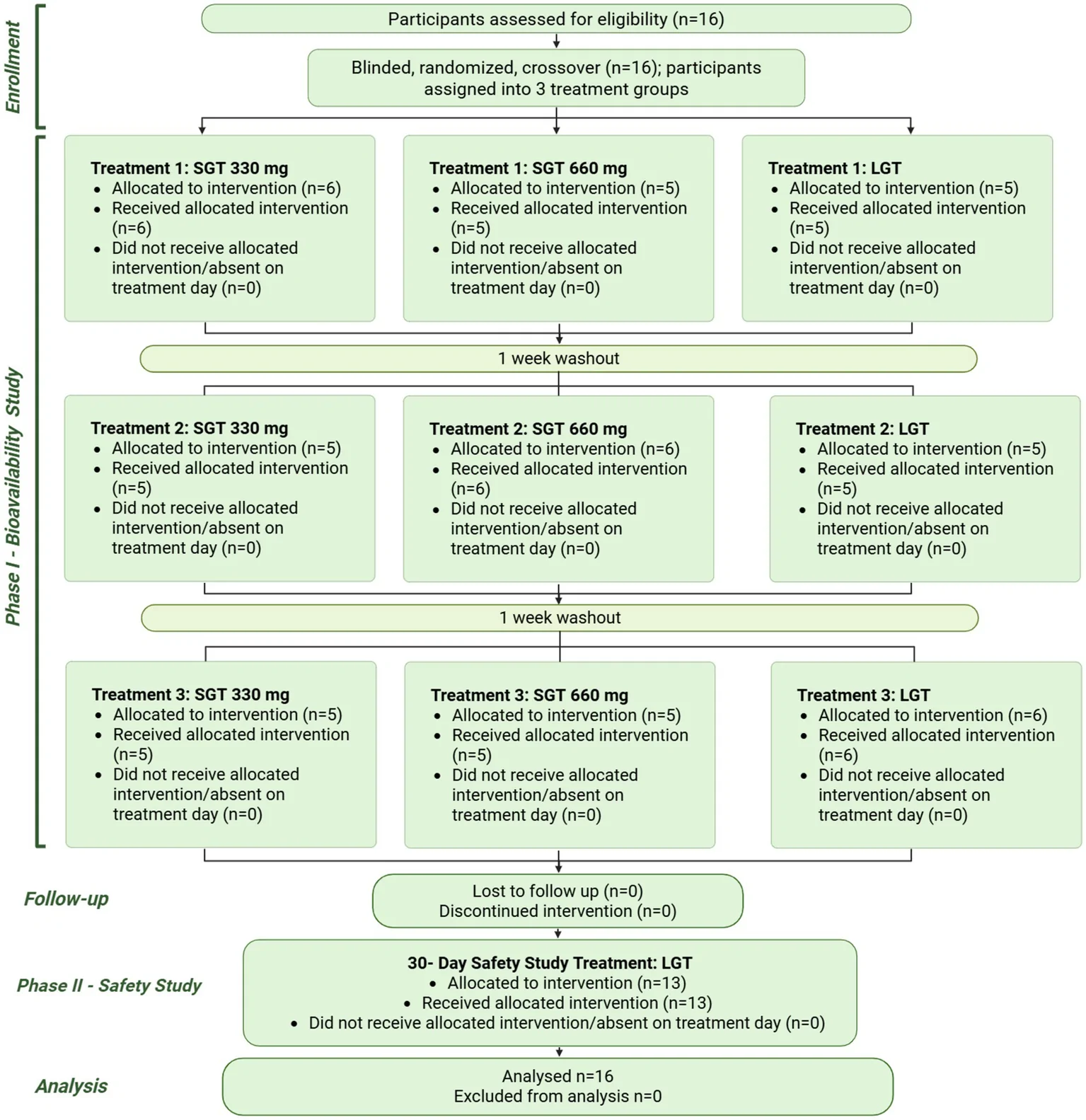
Flowchart showing the study design for Phase I (bioavailability study) and Phase II (safety study) involving 13 healthy adult participants. Created in BioRender. Ibi, A. (2026), https://BioRender.com/4o3u7nk.

The second part of the study was a 30-day open-label, single-arm safety assessment of LGT at a high daily dose of 1,320 mg (4 capsules/day). The goal was to evaluate the tolerability and safety of prolonged high-dose intake in healthy adults due to higher bioavailability. Thirteen participants from the bioavailability study continued into the safety phase (Figure 3). Routine blood chemistry markers were monitored biweekly, including routine blood chemistry parameters such as hepatic markers (e.g., AST, ALT), renal markers (creatinine, eGFR), lipid markers (HDL, LDL, total cholesterol, triglycerides), glucose and electrolytes. Capillary blood samples were collected biweekly, and participants completed weekly health questionnaires to monitor any adverse events.

### 2.6 Randomization and blinding

Block randomization with a 5:5:6 allocation ratio was performed by an independent study assistant not involved in data collection or analysis. Participants were randomly assigned to receive one of three green tea formulations, coded “A,” “B” and “C” referring to LGT 330 mg, SGT 330 mg and SGT 660 mg, respectively. There are six possible sequences with these treatments: ABC, ACB, BAC, BCA, CAB, CBA, which are listed twice in a Microsoft Excel spreadsheet to cover 12 participants. For the remaining 4 participants, there are six possible packs, each containing four different sequences that would result in a 5:5:6 allocation ratio when combined with the previous 12 sequences. A “RANDBETWEEN(1,6)” function was used to randomly select one of the six packs in Microsoft Excel. The four sequences from the selected pack would be listed after the previous 12 sequences, resulting in a total of 16 sequences. Thereafter, using the “RAND()” function in Microsoft Excel, a random number was generated for each treatment sequence; the 16 sequences were then shuffled by sorting the random numbers (from smallest to largest). The shuffled sequences were then assigned to participants 1 through to 16. The assigned capsules were placed into identical pill cases labeled only with participant identifiers. The participants were not aware of the identities of the treatments they were receiving. Only the independent study assistant had access to the treatment allocation list. The study participants, study coordinator, and principal investigator were blinded to the treatment assignments throughout the study period.

### 2.7 Statistical analysis

Statistical analyses and figures were created using GraphPad Prism, version 11.0 (California, USA). A comparison of pharmacokinetics of green tea catechins between the different formulations was performed using a mixed-effects model within the ANOVA module, with treatment as a fixed effect and subject as a random effect; pairwise comparisons were adjusted with Tukey’s correction. The data from the safety blood chemistry and electrolytes tests were analyzed by Repeated-Measures ANOVA. Data were considered significant at *p* ≤ 0.05. Data are expressed as mean standard ± error of deviation (SD). Because EGCG was detectable at pre-dose in several treatment periods, likely reflecting residual background exposure and known intra-individual variability in catechin pharmacokinetics, baseline-adjusted metrics were prespecified to quantify the treatment-related increase in systemic exposure attributable to each intervention. Consequently, iAUC₀–₂₄ and iC_max_ were considered the most appropriate measures for comparing formulation-dependent differences in post-dose EGCG exposure. Between-occasion variability in human EGCG pharmacokinetic parameters has also been reported following repeated green-tea administration [M.-J. (29)]. Period-specific baseline-adjusted endpoints were therefore calculated to distinguish the post-dose concentration increment from variable pre-dose exposure. For each treatment period, each participant’s pre-dose EGCG concentration was subtracted from each post-dose concentration. Baseline-adjusted iAUC₀–₂₄ was calculated from the resulting concentration-time profile, with areas below baseline retained as negative contributions. Baseline-adjusted iC_max_ was defined as the maximum post-dose concentration increment above the period-specific pre-dose value (*iC_max_ = C_max, observed_ – C_0_*).

An exploratory one-sided post-hoc power sensitivity analysis was performed for the prespecified directional comparisons of LGT 330 mg versus SGT 330 mg and SGT 660 mg versus SGT 330 mg. The directional alternative specified greater baseline-adjusted EGCG exposure for the first treatment in each comparison. Power was calculated using participant-level raw paired differences, paired Cohen’s *d_z_*, *n* = 16, and one-sided *α* = 0.05. Primary statistical inference remained based on two-sided mixed-effects models and 95% confidence intervals.

### 2.8 Determination of green tea metabolites using LC-HRMS

Capillary whole blood samples were analyzed by Liquid Chromatography-High Resolution Mass Spectrometry (LC-HRMS). The capillary whole blood (200 μL) samples collected with potassium EDTA coated devices (Minivette, Sarstedt, Germany) were immediately frozen at −20 °C until sample processing. Samples were prepared and analyzed according to previously published methods (42, 43). Plasma was separated from the whole blood samples by centrifugation and were allowed to equilibrate at room temperature and then digested with 100 μL of β-glucuronidase (from *Helix pomatia*, Millipore-Sigma, ≥ 100,000 IU diluted to 330 IU) in a pH 5 buffer and incubated at 37–40 °C for one hour. For extraction, 300 μL of acetone was added into the sample solution and sonicated for one hour at 37–40 °C. Then sample tubes were centrifuged at 16,000 x g for 5 min at room temperature, and the supernatant was transferred to a 96-well microplate for LC-HRMS analysis. A Thermo Vanquish Ultra High-Performance Liquid Chromatography (UHPLC) system coupled to a Thermo Q Exactive Orbitrap Mass Spectrometer was used to analyze the samples. Liquid chromatography was carried out with a binary solvent gradient progressing from 10% B to 100% B in 4.0 min and equilibrated for 4.9 min before the next injection. The mobile phases were 0.5% formic acid in water in A and Methanol in B. An Acme Xceed C18, 100 mm x 2.1 mm, 1.9 μm UHPLC column (Phase Analytical Technology) was used to perform the separation at a flow rate of 400 μL/min.

The Orbitrap mass spectrometer was calibrated at a resolution of 70,000, with an accepted mass deviation range of ± 5.0 ppm. Calibration was performed using Thermo Pierce LTQ Velos ESI Positive Ion Calibration Solution and Thermo Piece ESI Negative Ion Calibration. To minimize interference from the sample matrix, data was collected at a resolution of 70,000 and at a quadrupole isolation window of 3.0 m/z. Octyl gallate, used as the internal standard, was detected as a protonated adduct with a mass of 281.1395 m/z. Catechin was detected at 289.0718 m/z, epicatechin sulfate was detected at 369.0286 m/z, methyl epicatechin sulfate was detected at 383.0442 m/z, methyl epicatechin gallate was detected at 455.0984 m/z, methyl epigallocatechin was detected at 319.0823 m/z, methyl epigallocatechin sulfate was detected at 399.0391 m/z.

Data acquisition was carried out using Thermo Xcalibur 5.0, and data analysis was performed with Thermo TraceFinder 5.0, using a default mass tolerance setting of 5.00 ppm. Green tea metabolites in capillary whole blood concentration were quantified using internal standard calibration with a 6-point calibration curve, employing catechin (certified Reference Material, secondary standard, Millipore Sigma) as the chemical standard. Validation of the LC-HRMS assay was performed in accordance with ICH M10 bioanalytical method validation principles as a partial validation. Selected validation performance metrics included calibration linearity over 1–100 ng/mL (mean R^2^ = 0.991), inter-run precision of 8–15% coefficient of variation (CV) across low, medium, and high quality control (QC) levels, reported accuracy deviations of 12.7% at the lowest spike and 18.2%/2.7% at medium/high QC, and carryover of 4.7% of the analyte lower limit of quantification (LLOQ) response. During study-sample analysis, two blank injections were included after the calibration sequence, with an additional blank injection between sample sets from different participants to minimize residual analyte transfer into subsequent injections.

### 2.9 Blood chemistry analysis

Capillary blood samples were analyzed using an SD-1 Auto Dry Biochemistry Analyzer (Seamaty Technology Co., Ltd., Chengdu, China) to assess several well-established safety markers, including liver and kidney function parameters, electrolytes, and other relevant indicators. On each test day, approximately 300 μL of capillary blood was collected from each participant into lithium-heparin-coated micro vials (Sarstedt AG & Co. KG, Nümbrecht, Germany, reference #: 20.1292.100) to prevent coagulation. All blood samples were analyzed immediately after collection to maintain sample integrity and minimize pre-analytical variability.

The glucose assay had a stated linear range of 2–25 mmol/L. The manufacturer specifies an absolute deviation of ±0.8 mmol/L at concentrations of 2–8 mmol/L and a relative deviation of ±10% at concentrations greater than 8–25 mmol/L.

LDL cholesterol was calculated by the analyzer rather than measured directly, using LDL=TC−HDL−TG/2.2 , with all concentrations expressed in mmol/L. At the concentrations observed in this study, the analyzer specifies a relative deviation of ±10% for total cholesterol concentrations of 3.0–9.0 mmol/L, an absolute deviation of ±0.25 mmol/L for HDL concentrations of 0.2–1.5 mmol/L, and an absolute deviation of ±0.20 mmol/L for triglyceride concentrations of 0.7–2.0 mmol/L. Small changes in calculated LDL were therefore interpreted in view of the combined analytical variability of these component measurements.

### 2.10 Adverse event reporting

Participants were instructed to document all adverse events (AE) that occurred during the treatment periods via online health questionnaires collected on a weekly basis and to report these (based on severity) immediately to the research team. Criteria for AE rating is as shown in Supplementary material S2.

## 3 Results

### 3.1 Baseline data

The baseline and demographic data are shown in Tables 1, 2. A total of 16 healthy individuals (38.4 ± 7.7 years) participated in the pharmacokinetics study, while the total number of participants in the safety study was 13.

**Table 1:** Baseline characteristics of pharmacokinetics study participants (*n* = 16).

Characteristic	Male	Female	Combined
*N*	7	9	16
Age (years)	45.7 ± 9.6	31.0 ± 5.8	38.4 ± 7.7
Weight (kg)	80.0 ± 14.5	59.2 ± 13.2	69.6 ± 13.9
Height (cm)	175.1 ± 5.8	164.2 ± 8.0	169.7 ± 6.9
BMI	26.0 ± 3.6	22.0 ± 3.1	24.0 ± 3.4

**Table 2:** Baseline characteristics of safety study participants (*n* = 13).

Characteristic	Male	Female	Combined
*N*	5	8	13
Age (years)	37.0 ± 15.2	40.5 ± 10.0	39.2 ± 11.8
Weight (kg)	72.6 ± 14.6	67.0 ± 17.4	69.2 ± 16.0
Height (cm)	173.2 ± 8.4	165.8 ± 6.4	168.7 ± 7.8
BMI	24.1 ± 4.1	24.1 ± 4.7	24.1 ± 4.3

### 3.2 Pharmacokinetics

Total catechin metabolites (epicatechin gallate, epicatechin gallate sulfate, epigallocatechin gallate sulfate, methyl epicatechin sulfate, methyl epicatechin gallate sulfate, methyl epigallocatechin sulfate, and EGCG) were summed as an exploratory index of circulating green tea-derived compounds. Following administration of the green tea formulations, total circulating green tea metabolites increased rapidly. LGT produced numerically higher AUC₀–₂₄ (16,400 ± 14,100 ng·h/mL) and C_max_ (1,240 ± 850 ng/mL) than dose-matched SGT 330 mg, although these differences were not statistically significant (Figure 4; Table 3). Compared with SGT 660 mg, LGT showed lower total metabolite C_max_ (*p* = 0.002), consistent with the lower administered green tea extract dose. LGT also exhibited a numerically shorter T_max_ (2.8 ± 1.8 h). No significant differences were observed for T_max_, half-life (T_½_), or mean residence time (MRT) (*p* > 0.05 for all parameters).

**Figure 4:**
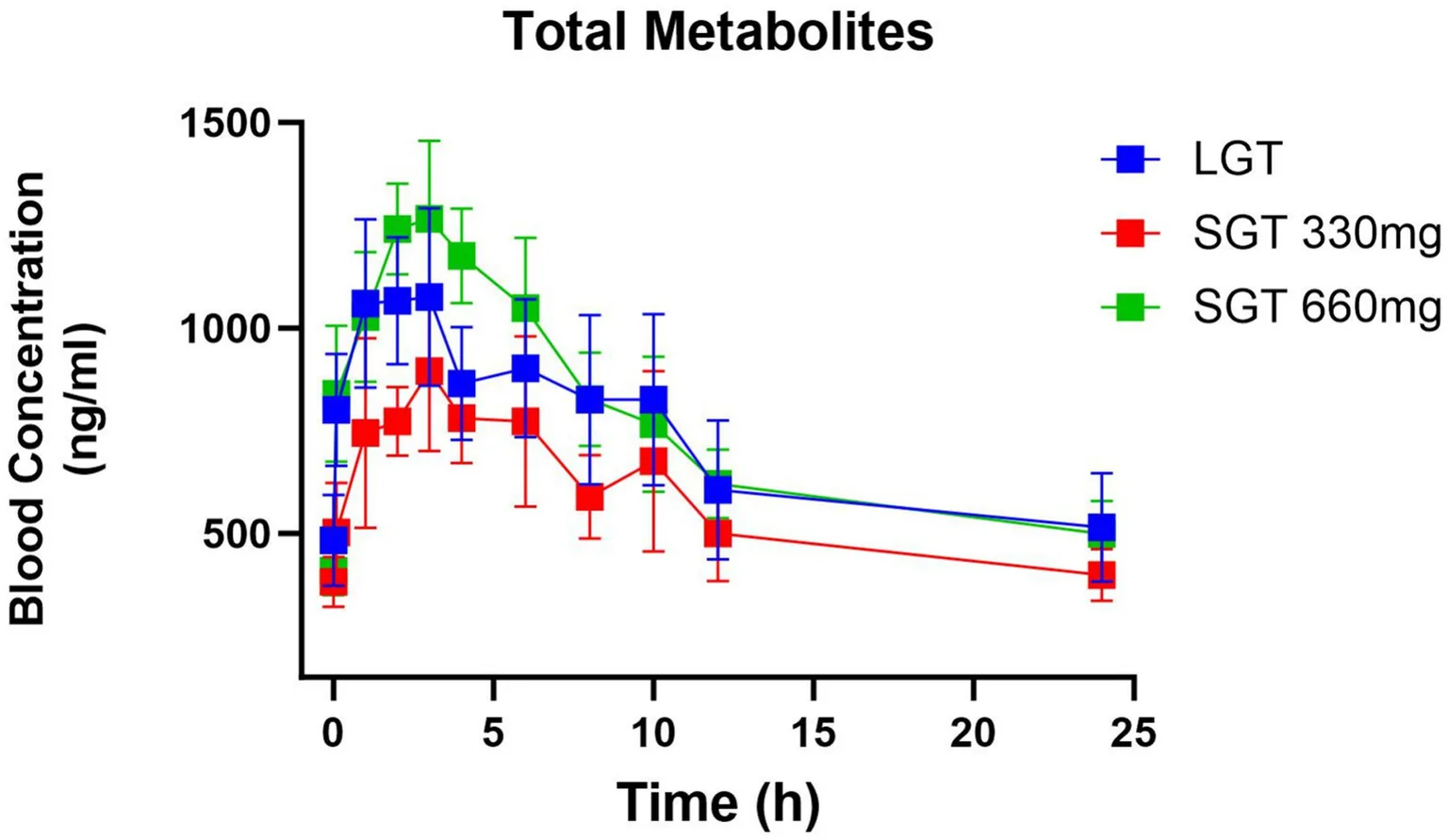
Blood concentration of total green tea metabolites over 24 h. Total green tea metabolites are the sum of catechin, epicatechin gallate, epicatechin gallate sulfate, epigallocatechin gallate sulfate, methyl epicatechin sulfate, methyl epicatechin gallate sulfate, methyl epigallocatechin sulfate, and EGCG. *Denotes *p* ≤ 0.05 between treatments using one-way repeated-measures ANOVA with Tukey’s multiple comparisons correction.

**Table 3:** Pharmacokinetics of total metabolites over 24 h.

Pharmacokinetic parameter	SGT 330 mg	SGT 660 mg	LGT 330 mg	*p*-value of LGT vs SGT 330 mg	*p*-value of LGT vs SGT 660 mg
AUC_0-24_ (ng·h/mL)	13,400 ± 10,400	17,700 ± 7,400	16,400 ± 14,100	0.965	0.498
C_max_ (ng/mL)	1,033 ± 850	1,470 ± 710	1,240 ± 850	0.250	0.002 *
T_1/2_ (h)	27.6 ± 21.9	32.9 ± 24.9	20.4 ± 16.9	0.316	0.994
T_max_ (h)	4.4 ± 5.3	3.0 ± 1.5	2.8 ± 1.8	0.347	0.753
MRTi (h)	40.8 ± 31.2	47.2 ± 34.9	30.2 ± 23.1	0.331	0.554

Data are presented as mean ± SD. Statistical analysis was performed by using One-way repeated measures ANOVA followed by Tukey’s correction. *p*-values show the statistical difference between LGT and SGT. *Denotes *p* ≤ 0.05 between treatments where *p* < 0.05 indicates significant difference.

Concentration-time profiles for individual catechins and their metabolites are shown in Supplementary Figure 1. For most of the metabolites, pharmacokinetic parameters did not differ significantly between treatments (Tables 4–10).

**Table 4:** Pharmacokinetics of EGCG over 24 h.

Pharmacokinetic parameter	SGT 330 mg	SGT 660 mg	LGT 330 mg	*p*-value of LGT vs SGT 330 mg	*p*-value of LGT vs SGT 660 mg
AUC_0-24_ (ng·h/mL)	6,427 ± 3,469	6,780 ± 4,214	7,229 ± 5,034	0.068	0.099
iAUC_0-24_ (ng·h/mL)	443 ± 1,570	2,200 ± 1921	1857 ± 1,479	0.032 *	0.796
C_max_ (ng/mL)	331 ± 188	421 ± 252	474 ± 342	0.031 *	0.575
iC_max_ (ng/mL)	88.6 ± 60.0	205 ± 135	267 ± 235	0.037 *	0.722
T_1/2_ (h)	58.0 ± 65.4	53.3 ± 60.6	33.8 ± 30.8	0.549	0.274
T_max_ (h)	6.81 ± 5.50	5.16 ± 3.58	3.94 ± 2.67	0.049*	0.864
MRTi (h)	85.3 ± 94.2	78.0 ± 87.6	50.0 ± 43.7	0.527	0.300

Data are presented as mean ± SD. Statistical analysis was performed using a mixed-effects model within one-way repeated-measures ANOVA followed by Tukey’s correction. *p*-values show the statistical difference of LGT versus the other formulations. *Denotes *p* ≤ 0.05 between treatments where *p* < 0.05 indicates significant difference. Raw AUC₀–₂₄ and C_max_ were natural-log transformed for inferential analysis. Baseline-adjusted iAUC₀–₂₄ and iC_max_ were analyzed on the raw scale; detailed raw-scale paired effect sizes and post-hoc power sensitivity analyses for iAUC₀–₂₄ and iC_max_ are provided in Supplementary material S3.

**Table 5:** Pharmacokinetics of epicatechin gallate over 24 h.

Pharmacokinetic parameter	SGT 330 mg	SGT 660 mg	LGT 330 mg	*p*-value of LGT vs SGT 330 mg	*p*-value of LGT vs SGT 660 mg
AUC_0-24_ (ng·h/mL)	1,280 ± 1,110	1910 ± 1,350	670 ± 390	0.585	0.051
C_max_ (ng/mL)	175 ± 153	199 ± 113	105 ± 69	0.843	0.169
T_1/2_ (h)	29.6 ± 28.2	115.2 ± 174.2	52.5 ± 62.6	0.963	0.885
T_max_ (h)	3.9 ± 2.3	6.5 ± 6.7	2.5 ± 2.2	0.628	0.141
MRTi (h)	44.2 ± 38.5	183.8 ± 263.1	74.2 ± 89.2	0.975	0.881

Data are presented as mean ± SD. Statistical analysis was performed by using mixed-effects model within One-way repeated measures ANOVA followed by Tukey’s correction. *p*-values show the statistical difference of LGT vs. other formulations. *Denotes *p* ≤ 0.05 between treatments where *p* < 0.05 indicates significant difference.

**Table 6:** Pharmacokinetics of epicatechin gallate sulfate over 24 h.

Pharmacokinetic parameter	SGT 330 mg	SGT 660 mg	LGT 330 mg	*p*-value of LGT vs SGT 330 mg	*p*-value of LGT vs SGT 660 mg
AUC_0-24_ (ng·h/mL)	1487.6 ± 901.4	1502.3 ± 883.8	1670.8 ± 1914.1	0.794	0.791
C_max_ (ng/mL)	139.6 ± 56.4	158.1 ± 79.1	138.2 ± 107.9	0.461	0.444
T_1/2_ (h)	25.1 ± 40.6	38.3 ± 71.7	25.0 ± 38.6	0.995	0.886
T_max_ (h)	3.6 ± 1.6	2.9 ± 1.7	5.1 ± 5.7	0.951	0.430
MRTi (h)	37.3 ± 59.3	54.4 ± 103.5	35.5 ± 55.6	0.988	0.968

Data are presented as mean ± SD. Statistical analysis was performed by using mixed-effects model within One-way repeated measures ANOVA followed by Tukey’s correction. *p*-values show the statistical difference of LGT vs. other formulations. *Denotes *p* ≤ 0.05 between treatments where *p* < 0.05 indicates significant difference.

**Table 7:** Pharmacokinetics of epigallocatechin gallate sulfate over 24 h.

Pharmacokinetic parameter	SGT 330 mg	SGT 660 mg	LGT 330 mg	*p*-value of LGT vs SGT 330 mg	*p*-value of LGT vs SGT 660 mg
AUC_0-24_ (ng·h/mL)	8,730 ± 16,180	4,594 ± 6,570	7,903 ± 15,148	0.996	0.974
C_max_ (ng/mL)	541 ± 1,021	492 ± 932	577 ± 915	0.943	0.991
T_1/2_ (h)	127 ± 199	36.6 ± 24.2	525 ± 1,478	0.999	0.929
T_max_ (h)	4.6 ± 3.9	3.2 ± 2.9	4.5 ± 4.1	0.995	0.559
MRTi (h)	184.8 ± 286.3	53.6 ± 35.6	759.5 ± 2133.1	0.999	0.913

Data are presented as mean ± SD. Statistical analysis was performed by using mixed-effects model within One-way repeated measures ANOVA followed by Tukey’s correction. *p*-values show the statistical difference of LGT vs. other formulations. *Denotes *p* ≤ 0.05 between treatments where *p* < 0.05 indicates significant difference.

**Table 8:** Pharmacokinetics of methyl epicatechin sulfate over 24 h.

Pharmacokinetic parameter	SGT 330 mg	SGT 660 mg	LGT 330 mg	*p*-value of LGT vs SGT 330 mg	*p*-value of LGT vs SGT 660 mg
AUC_0-24_ (ng·h/mL)	1,485 ± 560	2,710 ± 1720	1725 ± 1,159	0.898	0.032*
C_max_ (ng/mL)	293 ± 182	445 ± 187	457 ± 457	0.193	0.673
T_1/2_ (h)	13.6 ± 12.9	16.0 ± 23.5	6.5 ± 7.6	0.125	0.092
T_max_ (h)	2.5 ± 0.7	2.9 ± 2.7	1.3 ± 0.8	0.001*	0.048*
MRTi (h)	15.5 ± 13.6	19.7 ± 31.0	8.8 ± 8.5	0.221	0.149

Data are presented as mean ± SD. Statistical analysis was performed by using mixed-effects model within One-way repeated measures ANOVA followed by Tukey’s correction. *p*-values show the statistical difference of LGT vs. other formulations. *Denotes *p* ≤ 0.05 between treatments where *p* < 0.05 indicates significant difference.

**Table 9:** Pharmacokinetics of methyl epicatechin gallate sulfate over 24 h.

Pharmacokinetic parameter	SGT 330 mg	SGT 660 mg	LGT 330 mg	*p*-value of LGT vs SGT 330 mg	*p*-value of LGT vs SGT 660 mg
AUC_0-24_ (ng·h/mL)	913 ± 546	1,450 ± 760	1,070 ± 1,040	0.983	0.263
C_max_ (ng/mL)	87.0 ± 39.6	132.7 ± 50.8	107.6 ± 98.4	0.982	0.273
T_1/2_ (h)	21 ± 27	20 ± 16	16 ± 11	0.895	0.454
T_max_ (h)	3.9 ± 0.3	3.6 ± 1.5	2.6 ± 1.0	0.134	0.104
MRTi (h)	30.0 ± 35.9	27.0 ± 22.6	22.0 ± 14.6	0.823	0.544

Data are presented as mean ± SD. Statistical analysis was performed by using mixed-effects model within One-way repeated measures ANOVA followed by Tukey’s correction. *p*-values show the statistical difference of LGT vs. other formulations. *Denotes *p* ≤ 0.05 between treatments where *p* < 0.05 indicates significant difference.

**Table 10:** Pharmacokinetics of methyl epigallocatechin sulfate over 24 h.

Pharmacokinetic parameter	SGT 330 mg	SGT 660 mg	LGT 330 mg	*p*-value of LGT vs SGT 330 mg	*p*-value of LGT vs SGT 660 mg
AUC_0-24_ (ng·h/mL)	209 ± 58	320 ± 173	280 ± 161	0.853	0.812
C_max_ (ng/mL)	31.2 ± 8.7	45.7 ± 19.2	37.1 ± 14.6	0.749	0.547
T_1/2_ (h)	12.0 ± 10.4	7.9 ± 5.9	16.1 ± 11.0	0.424	0.099
T_max_ (h)	2.8 ± 0.8	2.4 ± 1.0	2.0 ± 0.9	0.048 *	0.047 *
MRTi (h)	18.4 ± 14.4	12.7 ± 7.9	23.5 ± 15.3	0.518	0.135

Data are presented as mean ± SD. Statistical analysis was performed by using mixed-effects model within One-way repeated measures ANOVA followed by Tukey’s correction. *p*-values show the statistical difference of LGT vs. other formulations. *Denotes *p* ≤ 0.05 between treatments where *p* < 0.05 indicates significant difference.

EGCG exhibited formulation-dependent differences (Table 4). Compared with dose-matched SGT 330 mg, LGT produced numerically higher raw EGCG AUC₀–₂₄ (7,229 ± 5,034 vs. 6,427 ± 3,469 ng·h/mL), although this difference did not reach statistical significance (*p* = 0.068). In contrast, baseline-adjusted EGCG exposure was significantly greater with LGT, with higher iAUC₀–₂₄ than with SGT 330 mg (1857 ± 1,479 vs. 443 ± 1,570 ng·h/mL; *p* = 0.032). LGT also produced significantly higher EGCG C_max_ (474 ± 342 vs. 331 ± 188 ng/mL; *p* = 0.031) and iC_max_ (267 ± 235 vs. 88.6 ± 60.0 ng/mL; *p* = 0.037). EGCG T_max_ was shorter with LGT than with SGT 330 mg (3.94 ± 2.67 vs. 6.81 ± 5.50 h; *p* = 0.049), consistent with earlier peak systemic exposure.

No significant differences in AUC₀–₂₄ and C_max_ were observed for the other metabolites (Tables 5–10). T_max_ for methyl epicatechin sulfate was shorter with LGT (1.3 ± 0.8 h) than with SGT 330 mg (2.5 ± 0.7 h; *p* = 0.001) and SGT 660 mg (2.9 ± 2.7 h; *p* = 0.048). Methyl epicatechin sulfate AUC was lower with LGT than with SGT 660 mg (*p* = 0.032). For methyl epicatechin gallate sulfate, LGT exhibited a numerically shorter T_max_ than the other formulations, although the differences did not reach statistical significance (*p* = 0.055). For methyl epigallocatechin sulfate, T_max_ following LGT administration was significantly shorter (2.0 ± 0.9 h) compared with both SGT 330 mg (2.8 ± 0.8 h; *p* = 0.048) and SGT 660 mg (2.4 ± 1.0 h; *p* = 0.047). Half-life was numerically longer with LGT (16.1 ± 11.0 h) than with SGT 330 mg (12.0 ± 10.4 h) and SGT 660 mg (7.9 ± 5.9 h), although these differences were not statistically significant.

### 3.3 Safety study

#### 3.3.1 Blood chemistry

As shown in Tables 11, 12, no statistically significant changes in blood chemistry or electrolyte values were observed over 30 days. Most values remained within reference ranges. In male participants (Table 11), mean glucose increased from 5.5 to 6.4 mmol/L, triglycerides increased from 2.4 to 3.0 mmol/L, and HDL decreased from 1.2 to 0.9 mmol/L; these changes were not statistically significant. Mean calculated LDL cholesterol in female participants changed from 3.3 ± 0.7 mmol/L at baseline to 3.4 ± 0.8 mmol/L after 30 days. The change was not statistically significant (*p* = 0.063). Because LDL was derived from TC, HDL, and TG measurements, the observed mean difference of 0.10 mmol/L cannot be distinguished confidently from analytical and biological variability. The increase in mean glucose in males exceeded the upper reference limit at Day 30; although not statistically significant, this finding should be interpreted cautiously in light of the small male subgroup sample size (*n* = 5), high variability, analytical variability, and absence of a control group.

**Table 11:** Blood chemistry and electrolytes data for male participants over 30 days (*N* = 5 males).

Males	Results (Mean ± SD)			*p*-value	Normal range
	Week 0	Week 2	30 Days		
ALT (U/L)	21.0 ± 7.7	29.4 ± 12.2	27.8 ± 10.6	0.111	9–50
AST (U/L)	27.2 ± 12.9	27.2 ± 6.4	24.2 ± 3.4	0.770	15–40
Total Bilirubin (μmol/L)	14.5 ± 4.1	19.3 ± 7.0	14.8 ± 7.4	0.140	3.4–21.0
Creatinine (μmol/L)	67.3 ± 8.9	64.9 ± 13.3	71.6 ± 7.6	0.156	44.0–97.0
Glucose (mmol/L)	5.5 ± 1.7	6.1 ± 1.4	6.4 ± 2.0	0.065	3.89–6.11
Total CO_2_ (mmol/L)	23.1 ± 0.6	21.7 ± 2.3	22.6 ± 0.2	0.333	22.0–29.0
Calcium (mmol/L)	2.4 ± 0.05	2.2 ± 0.6	2.4 ± 0.03	0.415	2.00–2.58
Phosphate (mmol/L)	1.1 ± 0.1	1.0 ± 0.3	1.2 ± 0.2	0.353	0.85–1.51
Magnesium (mmol/L)	0.9 ± 0.1	0.8 ± 0.3	1.1 ± 0.09	0.245	0.65–1.25
Sodium (mmol/L)	137.0 ± 2.7	138.4 ± 1.5	138.1 ± 1.7	0.477	135–147
Potassium (mmol/L)	4.7 ± 0.3	4.8 ± 0.3	4.6 ± 0.2	0.525	3.4–5.3
Chloride (mmol/L)	104.2 ± 1.9	104.7 ± 1.9	105.1 ± 1.2	0.485	99.0–122.0
GFR (mL/min/1.73m^2^)	119.0 ± 13.9	124.8 ± 18.6	116.8 ± 13.1	0.087	≥ 60
Total Cholesterol (mmol/L)	5.3 ± 1.3	4.6 ± 2.2	5.1 ± 1.4	0.235	2.0–5.19
LDL (mmol/L)	3.0 ± 0.8	3.1 ± 0.8	2.8 ± 0.8	0.183	1.50–3.40
HDL (mmol/L)	1.2 ± 0.2	1.2 ± 0.4	0.9 ± 0.2	0.244	> 1.19
Triglycerides (mmol/L)	2.4 ± 0.9	2.2 ± 0.9	3.0 ± 1.5	0.135	< 2.21

*p*-values are calculated from Repeated-measures one-way ANOVA summary; *p* < 0.05 indicates significant difference. Data presented as mean ± SD.

**Table 12:** Blood chemistry and electrolytes data for female participants over 30 days (*N* = 8 females).

Females	Results (Mean ± SD)			*p*-value	Normal range
	Week 0	Week 2	30 Days		
ALT (U/L)	19.3 ± 6.8	18.3 ± 6.5	18.6 ± 6.4	0.619	7–40
AST (U/L)	19.4 ± 3.2	19.6 ± 2.2	17.3 ± 2.5	0.081	13–35
Total Bilirubin (μmol/L)	9.5 ± 3.7	8.6 ± 3.3	10.6 ± 2.5	0.349	3.4–21.0
Creatinine (μmol/L)	56.3 ± 8.6	53.0 ± 10.6	53.6 ± 10.0	0.348	35–80
Glucose (mmol/L)	5.1 ± 0.7	5.2 ± 0.43	5.4 ± 0.4	0.392	3.89–6.11
Total CO_2_ (mmol/L)	21.9 ± 0.9	21.7 ± 1.3	21.9 ± 0.9	0.579	22.0–29.0
Calcium (mmol/L)	2.4 ± 0.06	2.3 ± 0.1	2.4 ± 0.07	0.0894	2.00–2.58
Phosphate (mmol/L)	1.1 ± 0.1	1.1 ± 0.1	1.1 ± 0.1	0.948	0.85–1.51
Magnesium (mmol/L)	0.9 ± 0.1	0.9 ± 0.1	0.9 ± 0.1	0.593	0.65–1.25
Sodium (mmol/L)	139.2 ± 2.0	137.8 ± 2.7	140.7 ± 3.3	0.162	135–147
Potassium (mmol/L)	4.8 ± 0.3	4.7 ± 0.4	4.8 ± 0.3	0.343	3.4–5.3
Chloride (mmol/L)	105.7 ± 1.2	104.5 ± 4.6	106.6 ± 1.3	0.336	99.0–122.0
GFR (mL/min/1.73m^2^)	120.2 ± 19.2	117.0 ± 9.7	114.8 ± 11.9	0.504	≥ 60
Total Cholesterol (mmol/L)	5.3 ± 0.8	5.1 ± 0.6	5.3 ± 0.9	0.076	2.0–5.19
LDL (mmol/L)	3.3 ± 0.7	3.2 ± 0.6	3.4 ± 0.8	0.063	1.50–3.40
HDL (mmol/L)	1.4 ± 0.2	1.3 ± 0.1	1.4 ± 0.1	0.118	> 1.19
Triglycerides (mmol/L)	1.2 ± 0.4	1.3 ± 0.5	1.2 ± 0.4	0.323	< 2.21

*p*-values are calculated from Repeated-measures one-way ANOVA summary; *p* < 0.05 indicates significant difference. Data presented as mean ± SD.

#### 3.3.2 Adverse events

Adverse event data from weekly questionnaires are summarized in Table 13. Mild, transient gastrointestinal symptoms were reported by two participants (≈15%), including heartburn, bloating, abdominal discomfort, and nausea. One participant experienced mild symptoms (heartburn, nausea, dizziness) during the first week. Another participant reported intermittent moderate gastrointestinal discomfort, including bloating and heartburn, with one episode rated as severe by self-report during week 2. All episodes reports reported by both participants were transient, resolving spontaneously or with food intake and required no medical intervention. Neither participant required treatment discontinuation or withdrawal, and the events did not meet the criteria for a serious adverse event. All events were transient and not considered related to the study product by the investigators, and none affected study adherence.

**Table 13:** Summary of adverse events reported during safety study.

Adverse events	Week 1	Week 2	Week 3	Week 4
Total number of participants (*n*) = 13				
Participants reporting AE (*n*)	1 (8%)	1 (8%)	1 (8%)	1 (8%)
Participants reporting type of AE (*n*)				
Symptoms				
Bloating	0 (0%)	0 (0%)	1 (8%)	1 (8%)
Constipation	0 (0%)	0 (0%)	0 (0%)	0 (0%)
Diarrhea	0 (0%)	0 (0%)	0 (0%)	0 (0%)
Heartburn	1 (8%)	1 (8%)	1 (8%)	1 (8%)
Pain or cramps	1 (8%)	1 (8%)	1 (8%)	1 (8%)
Rash	0 (0%)	0 (0%)	0 (0%)	0 (0%)
Nausea	1 (8%)	0 (0%)	0 (0%)	0 (0%)
Dizziness	1 (8%)	0 (0%)	0 (0%)	0 (0%)
Blurred vision	0 (0%)	0 (0%)	0 (0%)	0 (0%)
Other (unrelated to treatment)	0 (0%)	0 (0%)	0 (0%)	0 (0%)
Total AE by severity (*n*)				
Mild	4	0	1	0
Moderate	0	1	2	3
Severe	0	1	0	0
Life-threatening	0	0	0	0

## 4 Discussion

This study demonstrates that LipoMicel^®^ GTNAC^®^ extract (LGT), a novel lipid-based formulation, significantly improves the baseline-adjusted systemic delivery of a key green tea catechin, i.e., EGCG, relative to a standard formulation. These findings directly address the well-documented bioavailability limitations of green tea catechins that have restricted their clinical application. Importantly, despite higher baseline-adjusted EGCG exposure, the formulation was well tolerated, without any significant changes observed in common clinical safety markers or reported adverse events over 30 days at a high daily dose (1,320 mg GTE), supporting its safety for short-term use.

A study by Chow et al. found that daily green tea supplementation for 4 weeks in healthy adults was associated with either no adverse events or only mild gastrointestinal symptoms such as excess gas, upset stomach, nausea, heartburn, and abdominal discomfort (44). Similarly, two participants in the present study reported transient gastrointestinal symptoms. Although one heartburn episode was self-rates as severe, all events resolved without medical intervention, did not affect adherence, and were not considered serious adverse events. Oketch-Rabah et al. recommended avoiding green tea extracts on an empty stomach, suggesting that supplement tolerance may be influenced by meals (44).

The current 30-day safety study was open-label and single-arm, with safety laboratory values compared within participants against baseline. This design is consistent with an initial short-term tolerability assessment but limits causal interpretation of small metabolic shifts. In the absence of a placebo or active comparator group, it cannot be determined whether the observed, non-significant, changes reflect variability, dietary intake, lifestyle factors, measurement variability, the formulation, NAC, the lipid-based excipients or a combination of these factors. Therefore, these findings should be interpreted cautiously and confirmed in future controlled safety studies. Most blood chemistry and electrolyte values remained within reference ranges over the 30-day period of this study, even when LGT was given at a high dose. Small, non-significant increases in mean glucose among male participants and calculated LDL cholesterol among female participants were interpreted cautiously.

The summed total metabolites were an aggregate measure retained as an exploratory indicator of circulating catechin-related compounds whereas the primary interpretation of enhanced systemic exposure was focused on EGCG. Although total metabolite AUC did not differ significantly among treatments, LGT produced numerically greater peak concentrations than SGT 330 mg, with values that approached those observed with double-dose SGT. The principal evidence for enhanced systemic exposure, however, is based on EGCG. For EGCG, LGT produced significantly greater baseline-adjusted exposure than the equivalent dose of SGT, with 4.2-fold higher iAUC₀–₂₄ and 3.0-fold higher iC_max_. Raw EGCG AUC₀–₂₄ was numerically higher with LGT but did not reach statistical significance. This finding suggests that LipoMicel technology may substantially improve the absorption of intact EGCG, allowing a lower dose to achieve similar systemic exposure as a double dose of unformulated green tea extract. However, because fecal excretion, intestinal permeability, and gut microbiota-mediated metabolism were not assessed, the present study cannot determine the precise absorption mechanisms. Future studies should examine how formulation properties influence gastrointestinal handling, microbial metabolism, and catechin bioavailability.

In addition to improving EGCG exposure, LGT accelerated the appearance of methyl epicatechin sulfate in circulation, as evidenced by a significantly shorter T_max_ compared with both unformulated green tea treatments (LGT T_max_ 1.3 h vs. SGT 330 mg T_max_ 2.5 h and SGT 660 mg T_max_ 2.9 h; *p* = 0.001 and *p* = 0.048, respectively). Furthermore, the T_max_ of methyl epigallocatechin sulfate following LGT administration was significantly shorter compared to SGT formulations of both doses (*p* = 0.048 and *p* = 0.047, respectively). Half-life was numerically longer with LGT, although this difference was not statistically significant. Additional studies would be required to elucidate the underlying mechanism. Though they did not reach statistical significance, the T_max_ of other analytes such as epicatechin gallate and methyl epicatechin gallate sulfate were also improved. This may suggest more rapid absorption and systemic availability of these metabolites. Faster absorption kinetics are consistent with the proposed mechanism of lipid-based micellar delivery systems, which enhance the solubilization and transport of poorly water-soluble polyphenols across the gastrointestinal barrier (45, 46). Most other catechin metabolites showed similar exposure profiles across treatments (Tables 5–10) indicating that the primary influence of the LipoMicel formulation occurs during the absorption phase rather than through modification of downstream metabolic pathways. Once absorbed, catechins appear to undergo comparable biotransformation regardless of formulation.

A novel aspect of these formulations is the inclusion of N-acetylcysteine (NAC). Although NAC was originally included for its antioxidant and safety-supportive properties, its physicochemical and biological effects may also facilitate absorption. NAC’s mucolytic activity can reduce intestinal mucus viscosity, improving micelle permeation and mucosal contact (47–49). Beyond its mucolytic role, NAC is a cysteine prodrug that can support glutathione synthesis and thereby modify cellular redox status (50). This may be relevant because catechins such as EGCG are prone to oxidation and can form reactive quinone-like species that either decompose or conjugate with thiols, including GSH; EGCG-GSH and EGCG-NAC conjugates have been described in experimental systems and could, in principle, provide chemical protection for EGCG (43, 51, 52). EGCG can also interact with glutathione- and thioredoxin-related pathways in experimental models (53, 54), raising the possibility that catechin-NAC combinations act through broader redox networks rather than selective modulation of a single metabolic target. However, direct evidence that NAC administration changes catechin stability, conjugation, absorption, or metabolism in humans is lacking. Because the present study did not measure enzyme activity, transporter function, GSH- or NAC-derived catechin conjugates, GSH redox status, or redox-signaling endpoints, these proposed mechanisms remain hypothetical. NAC’s ability to support endogenous antioxidant defenses may nevertheless complement the intrinsic antioxidant properties of green tea catechins (55–58), making this a potentially useful formulation strategy that warrants direct mechanistic evaluation.

This study has several limitations. The relatively short duration limits assessment of long-term safety, tolerance, and sustained bioavailability patterns. The small sample size (*n* = 16) and inclusion of only healthy adults restrict generalizability, as variability in metabolic health, diet, and genetic background may influence catechin absorption and metabolism (59–62). The small sample size and high inter-individual pharmacokinetic variability resulted in limited statistical power and wide confidence intervals for the EGCG treatment effects. Although the crossover design reduced between-participant variability, the small sample and marked within- and between-participant EGCG variability produced imprecise estimates for several comparisons. The post-hoc power analysis was conditional on the observed effects. Therefore, the findings should be interpreted as preliminary and require confirmation in a larger, prospectively powered crossover study. The absence of a placebo group in the safety study is another major limitation in interpreting the metabolic shifts observed in the blood chemistry results. These changes may reflect variability, dietary intake, lifestyle factors, measurement variability, the formulation, NAC, the lipid-based excipients or a combination of these factors. A future safety study design employing a placebo or active comparator group could aid in determining the causality of the metabolic shifts observed. In addition, high interindividual variability observed in pharmacokinetic parameters may underscore the need for replication in larger, more diverse populations. Nevertheless, the crossover design mitigated between-subject variability and supports the robustness of within-subject comparisons.

## 5 Conclusion

This study compared the pharmacokinetics, safety and tolerability of two green tea formulations in healthy adults. A single dose of LipoMicel^®^ GTNAC^®^ (LGT), a novel lipid-based formulation co-delivered with N-acetylcysteine, increased baseline-adjusted systemic exposure to the primary green tea catechin EGCG compared with dose-matched standard green tea extract (SGT). LGT produced significantly higher iAUC₀–₂₄ (approx. 4-fold) and iC_max_ (approx. 3-fold) for EGCG, along with higher raw C_max_ and a shorter T_max_, while raw AUC₀–₂₄ was numerically higher but did not reach statistical significance. In a 30-day high-dose safety evaluation, LGT was well tolerated, with no serious adverse events or clinically relevant changes in blood chemistry. Together, these findings suggest that the LipoMicel^®^ formulation provides a safe and promising strategy for improving systemic EGCG exposure. Further studies in larger and more diverse populations are warranted to confirm these results and explore potential functional health applications.

## Data Availability

The original contributions presented in the study are included in the article/Supplementary material, further inquiries can be directed to the corresponding author.
