# Supplementary material for: Enhanced bioavailability and short-term safety of a micellar green tea extract formulation co-delivered with N-acetylcysteine: a randomized crossover study in healthy adults
Source: Front Nutr. 2026 Sep 23;13:1940156. doi: 10.3389/fnut.2026.1940156 (PMC13643905; doi:10.3389/fnut.2026.1940156)
Supplement: Supplementary file 1 [file Supplementary_file_1.docx]

*Supplementary Material S1. Individual Blood Concentration of Catechin Metabolites*

a) b)

c) d)

e) f)

g)

Figure 1. Capillary whole blood concentration of green tea compounds and metabolites a) EGCG b) epicatechin gallate c) epicatechin gallate sulfate d) epigallocatechin gallate sulfate e) methyl epicatechin sulfate f) methyl epicatechin gallate sulfate g) methyl epigallocatechin sulfate over time. Data are presented as mean ± SEM.
