# Supplementary material for: Enhanced bioavailability and short-term safety of a micellar green tea extract formulation co-delivered with N-acetylcysteine: a randomized crossover study in healthy adults
Source: Front Nutr. 2026 Sep 23;13:1940156. doi: 10.3389/fnut.2026.1940156 (PMC13643905; doi:10.3389/fnut.2026.1940156)
Supplement: Supplementary file 2 [file Supplementary_file_2.docx]

Supplementary Materials S2. Adverse Events

Participants documented any adverse events that occurred during the treatment periods via weekly collected online health questionnaires using the criteria in Supplementary Table 1 below.

Supplementary Table 1. Grading criteria of questionnaire for potential adverse events that could occur for participants during treatment.

| **Adverse Event** | **Grade** | **Description** |
| --- | --- | --- |
| Bloating | 1 | No symptoms |
|  | 2 | Mild bloating not affecting daily activities (1-3 on a 1-10 scale) |
|  | 3 | Moderate bloating affecting daily activities, manageable with rest (4-6 on a 1-10 scale) |
|  | 4 | Severe bloating significantly impacting daily activities (7-9 on a 1-10 scale) |
|  | 5 | Life-threatening, requiring urgent intervention or hospitalization (10 on a 1-10 scale) |
|  |  |  |
| Constipation | 1 | No symptoms |
|  | 2 | Mild decrease in bowel movements, manageable with over-the-counter interventions (1-3 on a 1-10 scale) |
|  | 3 | Moderate decrease in bowel movements, requires prescription medication or dietary modifications (4-6 on a 1-10 scale) |
|  | 4 | Severe decrease in bowel movements, impacting daily activities, medical intervention required (7-9 on a 1-10 scale) |
|  | 5 | Life-threatening, requiring urgent intervention or hospitalization (10 on a 1-10 scale) |
|  |  |  |
| Diarrhea | 1 | No symptoms |
|  | 2 | Mild increase in stool frequency or change in consistency (1-3 on a 1-10 scale) |
|  | 3 | Moderate increase in stool frequency or change in consistency (4-6 on a 1-10 scale) |
|  | 4 | Severe increase in stool frequency or change in consistency (7-9 on a 1-10 scale) |
|  | 5 | Life-threatening, requiring urgent intervention or hospitalization (10 on a 1-10 scale) |
|  |  |  |
| Heartburn | 1 | No symptoms |
|  | 2 | Mild heartburn, easily manageable with over-the-counter antacids (1-3 on a 1-10 scale) |
|  | 3 | Moderate heartburn, requiring prescription medication for symptom control (4-6 on a 1-10 scale) |
|  | 4 | Severe heartburn, interfering with daily activities, medical intervention required (7-9 on a 1-10 scale) |
|  | 5 | Life-threatening, requiring urgent intervention or hospitalization (10 on a 1-10 scale |
|  |  |  |
| Pain, cramps or a knotted feeling in abdomen | 1 | No symptoms |
|  | 2 | Mild pain, easily tolerated and does not interfere with daily activities (1-3 on a 1-10 scale) |
|  | 3 | Moderate pain, requires over-the-counter pain medication for symptom control (4-6 on a 1-10 scale) |
|  | 4 | Severe pain, significantly impacting daily activities, may require prescription medication (7-9 on a 1-10 scale) |
|  | 5 | Life-threatening, requiring urgent intervention or hospitalization (10 on a 1-10 scale) |
|  |  |  |
| Rash | 1 | No symptoms |
|  | 2 | Mild rash, minimal or no itching, limited area affected (1-3 on a 1-10 scale) |
|  | 3 | Moderate rash, moderate itching, spreading to larger areas, affecting daily activities (4-6 on a 1-10 scale) |
|  | 4 | Severe rash with extensive itching, interfering significantly with daily activities (7-9 on a 1-10 scale) |
|  | 5 | Life-threatening, requiring urgent intervention or hospitalization (10 on a 1-10 scale) |
|  |  |  |
| Nausea | 1 | No symptoms |
|  | 2 | Mild nausea without vomiting, relieved by non-prescription interventions (1-3 on a 1-10 scale) |
|  | 3 | Moderate nausea with or without vomiting, requires prescription medication (4-6 on a 1-10 scale) |
|  | 4 | Severe nausea with or without vomiting, not responsive to standard antiemetic therapy (7-9 on a 1-10 scale) |
|  | 5 | Life-threatening, requiring urgent intervention or hospitalization (10 on a 1-10 scale) |
|  |  |  |
| Dizziness | 1 | No symptoms |
|  | 2 | Mild dizziness, transient and does not affect daily activities (1-3 on a 1-10 scale) |
|  | 3 | Moderate dizziness, intermittent episodes, may require intervention for symptom control (4-6 on a 1-10 scale) |
|  | 4 | Severe dizziness, persistent episodes, interfering significantly with daily activities (7-9 on a 1-10 scale) |
|  | 5 | Life-threatening, requiring urgent intervention or hospitalization (10 on a 1-10 scale) |
|  |  |  |
| Blurred Vision | 1 | No symptoms |
|  | 2 | Mild blurring of vision, minimal impact on visual acuity (1-3 on a 1-10 scale) |
|  | 3 | Moderate blurring of vision, noticeable impact on visual acuity, may require corrective measures (4-6 on a 1-10 scale) |
|  | 4 | Severe blurring of vision, significant impairment of visual acuity, medical intervention required (7-9 on a 1-10 scale) |
|  | 5 | Life-threatening, requiring urgent intervention or hospitalization indicated (10 on a 1-10 scale) |
