# Supplementary material for: Enhanced bioavailability and short-term safety of a micellar green tea extract formulation co-delivered with N-acetylcysteine: a randomized crossover study in healthy adults
Source: Front Nutr. 2026 Sep 23;13:1940156. doi: 10.3389/fnut.2026.1940156 (PMC13643905; doi:10.3389/fnut.2026.1940156)
Supplement: Supplementary file 3 [file Supplementary_file_3.docx]

Supplementary Materials S3

Table S2. Paired effect sizes used in the post-hoc power sensitivity analysis of baseline-adjusted EGCG exposure

| Endpoint | Comparison | $\boldsymbol{n}$ | Effect estimate, 95% CI | Mean paired difference | SD of paired differences | Cohen’s $\boldsymbol{d}_{\boldsymbol{z}}$ | One-sided post-hoc power |
| --- | --- | --- | --- | --- | --- | --- | --- |
| $\boldsymbol{iAU}\boldsymbol{C}_{\boldsymbol{0-24}}$, ng·h/mL | LGT 330 mg vs. SGT 330 mg | 16 | GMR 1.15 (0.59–2.24) | 1417.300 | 1922.311 | 0.737 | 0.878 |
| $\boldsymbol{iAU}\boldsymbol{C}_{\boldsymbol{0-24}}$, ng·h/mL | SGT 660 mg vs. SGT 330 mg | 16 | GMR 0.79 (0.45–1.39) | 1757.186 | 2571.066 | 0.683 | 0.832 |
| $\boldsymbol{i}\boldsymbol{C}_{\mathbf{max}}$, ng/mL | LGT 330 mg vs. SGT 330 mg | 16 | GMR 1.75 (0.91–3.34) | 176.338 | 245.547 | 0.718 | 0.863 |
| $\boldsymbol{i}\boldsymbol{C}_{\mathbf{max}}$, ng/mL | SGT 660 mg vs. SGT 330 mg | 16 | GMR 1.15 (0.66–2.01) | 116.766 | 143.289 | 0.815 | 0.928 |

Mean paired differences were calculated as the first treatment minus SGT 330 mg. Paired Cohen’s $d_{z}$was calculated as the mean participant-level paired difference divided by the SD of those differences. Post-hoc power was calculated in G*Power using a two-sided matched-pairs test with n=16 and α = 0.05. Because some baseline-adjusted incremental values were non-positive, the incremental endpoints were analyzed on the raw scale and were not natural-log transformed. The power analysis was conditional on the observed treatment effects and was considered supplementary rather than evidence of prospective design adequacy.
